# *Wolbachia* Endosymbionts of Fleas Occur in All Females but Rarely in Males and Do Not Show Evidence of Obligatory Relationships, Fitness Effects, or Sex-Distorting Manipulations

**DOI:** 10.3389/fmicb.2021.649248

**Published:** 2021-03-12

**Authors:** Ron Flatau, Michal Segoli, Hadas Hawlena

**Affiliations:** Mitrani Department of Desert Ecology, Swiss Institute for Dryland Environmental and Energy Research, The Jacob Blaustein Institutes for Desert Research, Ben-Gurion University of the Negev, Midreshet Ben-Gurion, Israel

**Keywords:** antibiotic treatment, arthropod symbiosis, experiment, fitness, fleas, persistence mechanisms, reproductive manipulations, *Wolbachia*

## Abstract

The widespread temporal and spatial persistence of endosymbionts in arthropod host populations, despite potential conflicts with their hosts and fluctuating environmental conditions, is puzzling. Here, we disentangled three main mechanisms that are commonly proposed to explain such persistence, namely, obligatory relationships, in which the host is fully dependent on its endosymbiont, fitness advantages conferred by the endosymbiont, and reproductive manipulations imposed by the endosymbiont. Our model system reflects an extreme case, in which the *Wolbachia* endosymbiont persists in all female flea hosts but rarely in male ones. We cured fleas of both sexes of *Wolbachia* but found no indications for either lower reproduction, offspring survival, or a change in the offspring sex ratio, compared to *Wolbacia*-infected fleas. These results do not support any of the suggested mechanisms. We highlight future directions to advance our understanding of endosymbiont persistence in fleas, as well as in other model systems, with extreme sex-differences in endosymbiont persistence. Insights from such studies are predicted to shed light on the evolution and ecology of arthropod-endosymbiont interactions in nature.

## Introduction

Highly prevalent and dense endosymbiont populations that persist in arthropod host populations, despite potential conflicts with their hosts and fluctuating environmental conditions, are widespread in nature (Feldhaar, 2011). Several mechanisms have been proposed to explain such high persistence levels. One possible explanation is related to the tendency of the endosymbiont-host relationships to evolve into a full dependence of the host on the endosymbiont for its survival and reproduction in the form of **obligatory relationships** (Moran et al., 2008; Ferrari and Vavre, 2011). However, endosymbionts may promote persistence even when the host is not fully dependent on them, for example, by providing **fitness advantages** to the host, e.g., nutrient supplementation or protection from enemies (Feldhaar, 2011; Su et al., 2013; Cao et al., 2019). A third possible group of mechanisms is related to **reproductive manipulations** imposed by the endosymbiont on the host’s reproduction to enhance its own transmission (Werren et al., 2008). Considering the important function that endosymbionts serve in determining the structure and performance of natural communities and their potential uses in biological control (Ahantarig and Kittayapong, 2011), understanding the relative roles that these mechanisms play in the persistence of arthropod-endosymbiont systems and identifying new potential mechanisms constitute major goals with applied aspects.

*Wolbachia* is among the most widespread bacterial endosymbionts in nature, infecting various arthropod and nematode species (Werren et al., 2008; Gerth et al., 2014). Some strains of *Wolbachia* exhibit **obligatory relationships** with their host, providing them with essential functions (Nikoh et al., 2014). For example, in bedbugs, *Wolbachia* is essential for the host’s growth and reproduction by providing B vitamins, which are deficient in their blood-based diet (Hosokawa et al., 2010). Other *Wolbachia* strains are involved in facultative relationships with their hosts, providing their host with **fitness advantages**, thereby enhancing their spread in the host population (Dean, 2006; Moran et al., 2008). For example, *Wolbachia* may enhance their host’s fitness through protection against pathogens (Hedges et al., 2008; Teixeira et al., 2008) and through nutritional advantages (Brownlie et al., 2009).

Finally, *Wolbachia* can spread and persist in the host population by manipulating their host’s reproduction to enhance the fitness of infected females. Such **reproductive manipulations** may include: (i) male-killing (MK), where infected males are eliminated, resulting in reduced competition for the surviving female progeny (Hurst and Jiggins, 2000; Werren et al., 2008); (ii) the induction of parthenogenesis (IP), where females produce daughters asexually (Ma and Schwander, 2017); (iii) the feminization of genetic males (MF) allowing them to produce eggs (Narita et al., 2011); and (iv) cytoplasmic incompatibility (CI), where the offspring of infected males and uninfected females fail to develop (or may develop into males in the case of arthropods with haplodiploid sex determination), thereby providing a reproductive advantage to infected females (Mouton et al., 2005; Werren et al., 2008).

To better understand endosymbiont persistence patterns and mechanisms in their host, we studied the fitness effects of the endosymbiont *Wolbachia* on their flea host *Synosternus cleopatrae*, which infests desert rodents. This system provides an important opportunity to test the universality of the above-suggested persistence mechanisms, as several lines of evidence suggest that it might differ from most previously documented cases. First, this is one of a few cases in which extreme sex-specific differences in endosymbiont persistence were documented over both time and space (see also Lo et al., 2006; Richardson et al., 2019). In fact, in natural populations, all female fleas in all sampling locations and times possess *Wolbachia* at high loads (3 × 10^5^ ± 2 × 10^5^), whereas from 0 to 54% of the males possess *Wolbachia* at a detectable level, with low infection loads (7 × 10^3^ ± 1 × 10^4^; Cohen et al., 2015; Flatau et al., 2018). Such extreme bias in the endosymbiont persistence pattern suggests that the two sexes are prone to divergent selection pressures (Richardson et al., 2019). Second, the near absence of *Wolbachia* in male fleas also excludes the possibility of *Wolbachia* being involved in **obligatory relationships**, at least regarding the males. Third, a previous study suggested the possibility of some negative fitness effects of *Wolbachia* on female fleas, as females with a relatively higher density of *Wolbachia* had a lower fitness, negating the possibility of *Wolbachia* spread in the population *via* **fitness advantages**. Nevertheless, these results were observational and could have been confounded with the female physiological age (Flatau et al., 2018). Finally, high *Wolbachia* persistence in females is also not likely to be explained by the occurrence of strong **reproductive manipulation** because the host species does not exhibit a female-biased sex ratio, reducing the possibility of strong sex-distorting manipulations such as MK, IP, or MF (Werren et al., 2008). In addition, the low occurrence and extremely low density of *Wolbachia* in male fleas (Flatau et al., 2018) suggest the lack of strong CI (but see Richardson et al., 2019).

To disentangle the occurrence of these effects, we cured fleas of both sexes of *Wolbachia* and assessed the reproductive success of female fleas in relation to their physiological age and *Wolbachia* presence. A failure of cured female fleas to survive and reproduce would support an **obligatory relationship**. A reduction in the fitness-correlative traits of the cured fleas would support the **fitness advantage** mechanism. A higher proportion of female offspring in *Wolbachia*-infected, compared to cured, fleas would support the occurrence of a **reproductive manipulation** inducing a female-biased sex ratio (MK, IP, or MF). In addition, we characterized the location of these endosymbionts within female fleas, as this may hint at their function. For example, endosymbiont occurrence in the gut and gut appendages may suggest a nutritional role (Ben-Yosef et al., 2020; Reis et al., 2020).

## Materials And Methods

### Experimental Design

**Figure 1:**
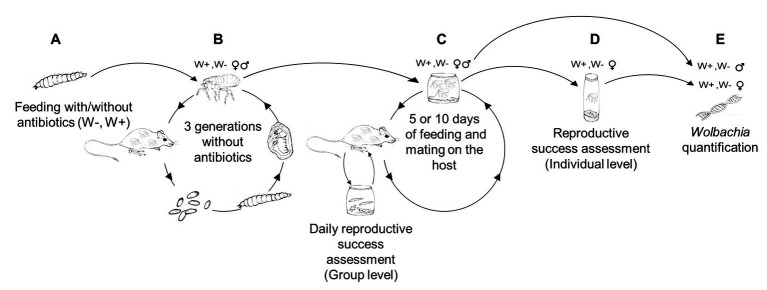
Experimental design. **(A)** Larval *Synosternus cleopatrae* fleas were randomly assigned to two treatment groups: a group that received antibiotics as a supplement to their food, *Wolbachia*-free (W^−^) and a control (W^+^) group. **(B)** Fleas from the two groups were reared on rodents for three additional generations without the antibiotic supplement. **(C)** Each treatment group was randomly divided into six subgroups, each subjected to either 5 or 10 days of feeding and mating. Specifically, the fleas were allowed to feed daily for 2 h on rodents and then collected and incubated overnight in separate boxes, where the average daily reproductive success of the group was assessed. **(D)** At the end of the three experimental stages, all female fleas were individually subjected to reproductive success assessments. **(E)** Then, a subset of fleas was subjected to *Wolbachia* quantification.

Larval *S. cleopatrae* fleas were randomly assigned to one of two treatment groups. One group received tetracycline antibiotics as a supplement to their food, and a control group received the same food except for the antibiotic supplementation (Figure 1A). Then, the adult fleas emerging from both treatment groups were reared separately on rodents for an additional three generations (150 days) without antibiotics (Figure 1B). The goal of this second stage was to reduce the direct effect of the antibiotics on the fleas and to allow the fleas to restore their natural bacterial community, excluding *Wolbachia* that cannot be acquired from the environment (Werren et al., 2008). The only other potential maternal transmitted bacteria that have been detected in *S. cleopatrae* fleas belong to *Rickettsia*. However, it was detected in one male out of 59 male and female fleas in one study (Cohen et al., 2015) and in one of 91 pools of fleas in another (Rzotkiewicz et al., 2015), suggesting that *Wolbachia* is the main intra-cellular endosymbiont found in these female fleas. For the third experimental stage, each treatment group was randomly divided into six subgroups (15 females and 15 males per group). Each subgroup was then subjected to either 5 or 10 days of feeding and mating. Specifically, the fleas were allowed to feed and mate on rodents for 2 h daily. The fleas were then collected and incubated overnight in separate boxes, where the average daily reproductive success of the entire subgroup was assessed (i.e., group level; Figure 1C). At the end of the third experimental stage, female fleas were collected into separate plastic vials, where the individual parent females were allowed to lay eggs for 24 h. Then, the vials were subjected to reproductive success assessments (i.e., individual level; Figure 1D), and a subset of the parental and offspring fleas were subjected to *Wolbachia* assessments (Figure 1E). Below, we detail each of the experimental stages.

### Establishment of the *Wolbachia*-Free (W^−^) and *Wolbachia*-Control (W^+^) Treatment Groups

One thousand *S. cleopatrae* fleas were randomly collected from a laboratory colony (courtesy of Prof. Krasnov, Ben-Gurion University). This flea colony has been maintained for 17 years (124 generations) on laboratory-reared gerbils, during which the genetic diversity was sustained through an annual mixing with wild fleas. Notably, although some genomic differences between laboratory and wild fleas are possible, the observed pattern of high *Wolbachia* prevalence (100%) and density in females compared to males is consistent in both populations (Flatau et al., 2018). These fleas were allowed to feed and mate on 10 *Gerbillus andersoni* rodents and to oviposit eggs in the rodent sterile sand bedding for 7 days. Then, the sand from all cages was mixed and equally divided into 10 20 cm × 15 cm × 10 cm boxes that were incubated at 25 ± 1°C and 95 ± 3% humidity in a growth chamber (I-41Nl, Percival Scientific, Inc.). Five of the boxes were randomly assigned to the antibiotic treatment, and the other five were used as controls. For the next 12 days, all boxes were supplemented every 4 days, with 10 g of larvae food mixed in the sand. In the treatment boxes, the food was mixed with an increasing amount of tetracycline powder (100, 200, and 300 mg). Since the tetracycline is fully degraded after 4 days at our incubation conditions, whereas the amount of food consumed by the flea larvae is negligible relative to the supplemented amount, this feeding regime resulted in a concentration of approximately 10 mg tetracycline per 1 g of food throughout the feeding period. Such concentration was found to be most efficient in *Wolbachia* clearance during preliminary trials comparing various combinations of antibiotic types and concentrations (Flatau, unpublished data). The food supplement was composed of 94% dry bovine blood, 5% millet flour and ground local vegetation, and 1% freshly ground rodent excrement. This supplement provides *ad libitum* food for the flea larvae, thus reducing intraspecific competition and increasing larval survival rates (Khokhlova et al., 2014).

### Feeding and Mating of Adult Fleas on Rodents

Daily feeding and mating of adult fleas took place on laboratory-reared *G. andersoni* rodents. Fleas were fed in groups of 30 (0.5 sex ratio) to reduce their negative impact on rodents and intraspecific competition among fleas (Hawlena et al., 2006, 2007). To prevent the rodent from grooming, which may harm the fleas, we placed each rodent inside a metal net tube that restricted their movement. Each flea group was fed for 2 h, and then the fleas were brushed off the rodent over a white plastic pan until all were recovered. Exceptions included four cases in which the flea could not be found, and hence its host was not reused, and the 21 cases in which the flea died during feeding. This feeding period is considered sufficient for the fleas to consume a full blood meal and mate (Khokhlova, personal comment). The fleas from each group were incubated overnight at 25 ± 1°C and 95 ± 3% in separately ventilated 250-ml plastic boxes embedded with 5 ml of sterile sand, where they could lay eggs. These plastic boxes were later used for the daily reproductive success assessment at the group level. Flea subgroups were rotated daily between individual rodents to maximize the number of rodents encountered by each group.

### Reproductive Success Assessment

The reproductive success of female fleas was evaluated at both the group and the individual female levels. For the group-level assessment, we used the 250-ml plastic boxes, in which the female fleas were incubated in groups overnight and where they laid eggs following the daily feeding and mating stage (Figure 1C). This procedure was repeated every day with a new plastic box, while the previous box was kept in incubation for 50 days, until all offspring have emerged. The boxes were filled with 5 ml of sterile sand and supplemented with 1.25 ml of antibiotic-free larval food (see “establishment of the *Wolbachia*-free and control groups”) and incubated at 25 ± 1°C and 95 ± 3% relative humidity. After each box reached 50 days of incubation, all emerged offspring were counted and sexed, and the daily average offspring number per female and offspring sex ratio of each group were quantified.

For the individual level assessment, 43 *Wolbachia*-free and 39 control female fleas were sampled on day 5 of the third experimental stage, following feeding. Then, 45 *Wolbachia*-free and 40 control female fleas were sampled on day 10 of the third experimental stage, following feeding. These 167 female fleas were placed into individual ventilated 10-ml plastic vials with 1 ml of sterile sand. Vials were supplemented with 0.25 ml of antibiotic-free larval food and incubated. From day 30 and on, the vials were monitored daily for newly emerged offspring. Emerged adults were collected into individual 1.5-ml Eppendorf tubes filled with 0.05 ml of sterile sand and incubated at 25 ± 1°C and 95 ± 3% relative humidity, where they were monitored daily until their death.

The emerged adults were not fed, and hence the number of days until their death was used to quantify their survival rate under starvation. Upon death, each offspring was stored in 70% ethanol at −80°C until it was sexed, measured, and, in some cases, subjected to *Wolbachia* quantification (stage E). For each offspring, we measured the two tibias (three repeated measurements per tibia), and the mean between them was raised to the power of three and used as an approximation of each offspring’s body size (Messika et al., 2017). Tibia measurements were performed with a stereomicroscope (SMZ18) equipped with a digital camera (DS-Fi2) and with the aid of the program NIS Elements Documentation (Nikon Instruments Inc.). Accordingly, we estimated the reproductive success (RS) of individual females using an integrated index, following Flatau et al. (2018):

Equation 1: $RS=\overset{NF}{\underset{i}{\sum }}\left(BS_{F}\times PS_{F}\right)+\overset{NM}{\underset{I}{\sum }}\left(BS_{M}\times PS_{M}\right)$, where NF and NM are the total numbers of female and male offspring, respectively, BSF and BSM are the body sizes of female and male offspring, respectively, and PSF and PSM are the survival probabilities of female and male offspring under starvation, respectively, estimated from the Kaplan-Meier survival analysis (packages “survival” and “survminer”; R Core Team, 2020).

### *Wolbachia* Quantification

DNA was extracted from all-female parent fleas, 40 of the parent males (10 per treatment-age combination), and 26 of the female offspring (13 per treatment group), using the QIAGEN DNeasy Blood and Tissue Kit and was subjected to quantitative polymerase chain reaction (qPCR) tests following the primers and conditions described in Flatau et al. (2018). In each extraction session, a negative control was added in which all the reagents were added to double distilled water instead of fleas. These control extracts were included in the qPCR runs and none of them were amplified.

### FISH Analyses

Fluorescence *in situ* hybridizations (FISH) analyses were used to confirm antibiotic supplementation efficiency and locate the *Wolbachia* cells in control fleas. Specifically, 20 female and 20 male fleas (10 per treatment) were immobilized at −20°C for 2 min and then dissected, and parts of their digestive systems (saliva glands, Malpighian tubules, and midguts) and reproductive organs (ovaries, spermathecae, and the male genitalia) were fixed, marked, and scanned, following Kliot et al. (2014). *Wolbachia*-free fleas and flea samples without probes were used as negative controls.

### Data Analysis

To test the effect of *Wolbachia* on flea reproductive success at the group level, we performed a generalized linear model, with the treatment (control “W^+^” or *Wolbachia*-free “W^−^”), female physiological age (covariant; 1–9 days), and the interaction between them as independent factors, and the number of offspring per female and the offspring sex ratio, as dependent variables.

To test the effect of *Wolbachia* on the female reproductive success at the individual level, we performed generalized linear mixed models, with the treatment (“W^+^” or “W^−^”), female physiological age (5 or 10 days), and the interaction between them as fixed factors, and the group of fleas with which they were daily fed together on a host, as a random factor. We conducted one analysis with the integrated reproductive success index as a dependent variable. We then added separate analyses to investigate the relative importance of different fitness components by considering the offspring development time from egg to emerging adult, the number of offspring per female, their body size, the sex ratio (proportion of females), and the probability of offspring survival under starvation, each separately as dependent variables.

To account for sexual polymorphism in development time and body size, male and female offspring were analyzed separately in the relevant analyses. To account for sexual polymorphism in the survival time of offspring (Krasnov, 2008), we standardized these values. This was done by converting the survival time of each offspring (e.g., a female that survived 18 days) to the probability of fleas from its own sex to survive until this day (e.g., probability of female fleas to survive 18 days), using the packages “survival” and “survminer” (R Core Team, 2020). For the analysis of the sex ratio that may largely depend on the total number of offspring per female, we added the offspring number as a covariant. All analyses were performed by using the GLM and GLMM packages lme4 (Bates et al., 2015) and lmerTest (Kuznetsova et al., 2017; R Core Team, 2020).

## Results

The tetracycline supplementation worked efficiently, as even after three antibiotic-free generations, there was no indication of *Wolbachia* in any of the treated fleas that were tested (*N* = 88 parental females, *N* = 13 female offspring, and *N* = 20 parental males). In contrast, as expected, all female fleas from the control group (*N* = 79 parental female and *N* = 13 female offspring) and six of the 20 parental control males were *Wolbachia*-positive. Consistent with the *Wolbachia* loads observed in wild fleas (Flatau et al., 2018), loads were significantly higher for control females than for control males (3 × 10^5^ ± 2 × 10^5^, for females, and 7 × 10^3^ ± 1 × 10^4^, for males).

**Figure 2:**
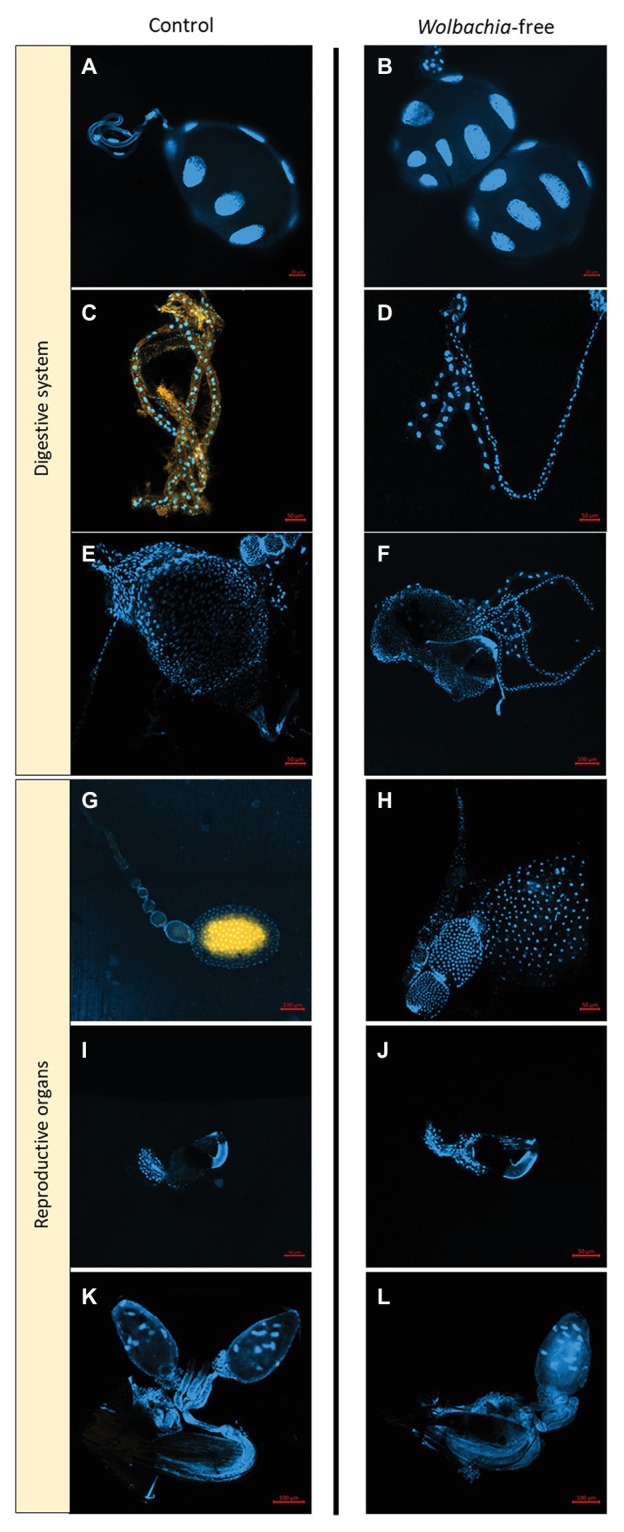
*Wolbachia* localization within *Synosternus cleopatrae* fleas. Fluorescence *in situ* hybridizations of organs in the ingestion and reproductive systems of control (W^+^, left) and *Wolbachia*-free (W^−^, right) fleas. **(A,B)** saliva gland, **(C,D)** Malpighian tubules, **(E,F)** midgut, **(G,H)** ovary, **(I,J)** spermatheca, and **(K,L)** male genitalia. DAPI stain for double-stranded DNA is indicated in blue and the specific probe for *Wolbachia* is indicated in yellow. For optimal illustration, except male genitalia, all other organs were taken from female fleas, although male fleas were also tested (see text).

*Wolbachia* presence in control female fleas and absence in female fleas of the *Wolbachia*-negative treatment were further confirmed by the FISH analyses (Figure 2). In control females, loads of *Wolbachia* cells were detected in the ovaries and Malpighian tubules, but not in the saliva gland, midgut, or spermatheca (Figure 2). No *Wolbachia* cells were detected in any of the male organs of either treatment groups. This failure to detect *Wolbachia* in any of the males including those that were not treated by antibiotics was possibly because *Wolbachia* titers were too low to be detected by the FISH protocol that we used (Schneider et al., 2018).

At the group level, we only found an age effect on the number of offspring per female, where the number of offspring increased with the parent female’s physiological age. Neither treatment, age, nor the interaction between the two had a significant effect on the offspring sex ratio (Table 1).

**Table 1:** Experimental results.

**Group-level analysis**					
**Dependent variable**	**W**^**+**^		**W**^**−**^	**F statistics**	
Mean offspring number per female	*Y* = −0.3 + 0.4X *R*^2^ = 0.8		*Y* = −0.3 + 0.5X *R*^2^ = 0.8	Age: 308^***^ Treatment: 1 Age × Treatment: 0.62	
Sex ratio	*Y* = 0.6 − 0.02X *R*^2^ = 0.04		*Y* = 0.5 − 0.002X *R*^2^ = 0.0007	Age: 0.8 Treatment: 0.04 Age × Treatment: 0.4	
**Individual-level analysis**					
**Dependent variable**	**W**^**+**^ **d 5**	**W**^**−**^ **d 5**	**W**^**+**^ **d 10**	**W**^**−**^ **d 10**	**T statistics**
Reproductive success index	13 ± 3	11 ± 3	20 ± 3	20 ± 3	Age: 3^*^ Treatment: 0.7 Age × Treatment: 0.5
Offspring number per female	2 ± 0.4	2 ± 0.4	3 ± 0.4	4 ± 0.4	Age: 2.8^*^ Treatment: 0.9 Age × Treatment: −0.9
Sex ratio	0.5 ± 0.05	0.5 ± 0.05	0.6 ± 0.05	0.5 ± 0.05	Age: −0.4 Treatment: 0.3 Age × Treatment: 0.6
Offspring survival	0.5 ± 0.04	0.6 ± 0.05	0.5 ± 0.04	0.5 ± 0.05	Age: −1 Treatment: −0.9 Age × Treatment: 0.8
Offspring male development	41 ± 0.3	42 ± 0.3	40 ± 0.3	40 ± 0.3	Age: −5^***^ Treatment: −2 Age × Treatment: 2
Offspring female development	34 ± 0.2	35 ± 0.3	32 ± 0.2	32 ± 0.2	Age: −4^**^ Treatment: −2 Age × Treatment: 1
Offspring male body size	7 ± 0.1	7 ± 0.3	7 ± 0.1	7 ± 0.1	Age: 0.2 Treatment: 0.1 Age × Treatment: −0.2
Offspring female body size	13 ± 0.1	13 ± 0.1	14 ± 0.1	13 ± 0.1	Age: 1 Treatment: −0.1 Age × Treatment: 0.3

Means ± standard errors of the fitness-correlated parameters assessed for Wolbachia-free (W^−^) and control (W^+^) female fleas at various ages (day 5 and day 10 correspond to age 5 days and age 10 days, respectively) by the group and individual level analyses. Statistical results are provided on the right side.

* *p < 0.05*;

** *p < 0.01*;

*** *p < 0.001*.

Similarly, at the individual level, the physiological age of the female parent significantly affected the integrated index of reproductive success, the number of offspring per female, and offspring developmental time (Table 1). In contrast to our predictions, there was also no effect of the treatment or treatment × age interaction on any of the dependent variables at this level.

## Discussion

Three main mechanisms are commonly proposed to explain the temporal and spatial persistence of arthropod endosymbionts in their host populations: **obligatory relationships**, in which the host is fully dependent on its endosymbiont, **fitness advantages** conferred by the endosymbiont, and **reproductive manipulations** imposed by the endosymbiont. To shed light on the universality of these mechanisms across host-endosymbiont systems, we experimentally explored the interactions between *Wolbachia* and their flea host, which at the first glance, seem to differ from those in most documented systems, due to the extremely high persistence levels over time and space in female, but not in male, hosts. Our results do not support any of the tested mechanisms, as neither fitness estimates nor sex ratio differed between control fleas and fleas cured of *Wolbachia.* Below, we discuss these results and highlight future directions required to better understand the interactions between *S. cleopatrae* fleas and their *Wolbachia* endosymbionts, as well as other systems with extreme sex-differences in endosymbiont persistence.

### Hypothesis 1 Is Not Supported: The Interaction With the Endosymbiont Is Not Obligatory for Either Female or Male Hosts

Our results refute this hypothesis as both female and male fleas were able to survive and reproduce for multiple generations without the presence of *Wolbachia*, confirming that the interaction is not obligatory for either sex. Hence, *Wolbachia* should be considered as a facultative endosymbiont in this system, and thus, its potential effects on the host can range from negative (Vorburger and Gouskov, 2011), through neutral (Moran et al., 2008), to positive (Brownlie et al., 2009; Vorburger and Gouskov, 2011).

Likewise, other model systems with potential sex-differences in endosymbiont persistence (as suggested by sex-differences in endosymbiont prevalence) seem to have facultative relationships with both female and male hosts. This is reflected by the occurrence of some female host individuals that did not carry the endosymbiont (e.g., *Rickettsia*-*Scymnus frontalis*, *Spiroplasma*-*Adalia punctate*, *Spiroplasma*-*Anisosticta punctate*, *Wolbachia*-*Coccidula rufa*, *Wolbachia*-*Rhyzobius litura*, *Wolbachia*-*Sogatella furcifera*, *Wolbachia*-*Ctenocephalides canis*, and “*Candidatus* Midichloria mitochondrii”-*Ixodes ricinus*; Noda et al., 2001; Gorham et al., 2003; Weinert et al., 2007; Sassera et al., 2008) or by the successful survival and reproduction of endosymbiont-cured hosts (e.g., *Wolbachia*-*Drosophila pseudotakahashii*, Richardson et al., 2019). Long-term field surveys are required to confirm whether the above-mentioned model systems indeed demonstrate sex-differences in endosymbiont persistence over time and space. If they do, these studies, taken together, may suggest that such a persistence pattern is not necessarily related to the obligatory dependence of the females on the endosymbiont.

### Hypothesis 2 Is Not Supported: There Is No Evidence for Fitness Advantages for Female Hosts Carrying the Endosymbiont

We found no support for this hypothesis, as there was no reduction in the reproductive success of females cured of *Wolbachia*, either when they were tested individually or in a group (Table 1). Investigations at the individual level allowed us to obtain a high-resolution snapshot view of *Wolbachia* effects on the current reproductive success of female fleas at early (5 days) and older ages (10 days), which are typically associated with low and high *Wolbachia* loads, respectively (Flatau et al., 2018). The group level complemented the individual level analysis by providing continuous information on daily reproduction in a more realistic social environment, including multiple female and male fleas.

Similar to our study, the only other two model systems with potential sex-differences in endosymbiont persistence, in which fitness effects were tested, failed to demonstrate differences between the reproductive success of control and endosymbiont-cured hosts (*Wolbachia* and *Sogatella furcifera* or *Drosophila pseudotakahashii*; Noda et al., 2001; Richardson et al., 2019). This may suggest that the high population persistence of endoparasites in female hosts may be maintained without obvious fitness advantages for the host.

The exploration of endosymbiont effects on its host fitness is almost a standard practice in symbiosis studies. This can be done either by relying on the natural variation in endosymbiont load while correlating it with host-fitness-related traits (e.g., Unckless et al., 2009; Segoli et al., 2013) or by directly manipulating endosymbiont presence *via* curing or infecting the host (Koga et al., 2007; Da et al., 2016; Karimi et al., 2019). Our results emphasize the limitations of the correlative approach, as when we previously adopted such an approach, we found potential evidence for *Wolbachia*-negative effects on *S. cleopatrae* females (Flatau et al., 2018). Such negative effects were not detected *via* experimental manipulation in the current study and, instead, were likely the result of a confounding effect between the flea age and *Wolbachia* load. Thus, we encourage researchers to face the challenges entailed by endosymbiont curing, endosymbiont injection, or both, to better characterize host-endosymbiont relationships.

Notably, our experiments were conducted under favorable laboratory conditions. To complement the view of *Wolbachia* fitness effects on its host, future experiments should be conducted under more demanding conditions, e.g., food limitation, high competition, and enemy presence (Brownlie et al., 2009; Gavotte et al., 2010; Vorburger and Gouskov, 2011), and on earlier developmental stages (Da et al., 2016). For example, Cao et al. (2019) found that *Wolbachia* provides fitness advantages to its *Drosophila simulans* host only when breeding on fungi-infected fruits. In another example, Brownlie et al. (2009) showed that *Wolbachia* infection confers a positive fecundity benefit for *D. melanogaster* only when they were reared on iron-restricted or overloaded diets.

In particular, several lines of evidence suggest potential fitness advantages that may be more pronounced in *S. cleopatrae* females than in male fleas under unfavorable conditions. First, although we have not quantified *Wolbachia* in flea eggs and larvae, the FISH analysis of control females suggests that all eggs carry *Wolbachia*. This implies that males may experience a secondary loss of infection during their development, which could be indicative of a facultative nutritional role of *Wolbachia*, since females have greater nutritional needs than males in blood sucking arthropods (e.g., Krasnov, 2008; Ben-Yosef et al., 2020). Second, the dominant occurrence of *Wolbachia* in the Malpighian tubules of *S. cleopatrae* females (Figure 2) may further support a nutritional role (Ben-Yosef et al., 2020; Reis et al., 2020) or indicate that these organs may store *Wolbachia* for other beneficial functions (Faria and Sucena, 2013). Altogether, this evidence may suggest that under restricted conditions, e.g., low nutrient availability for the larvae, anemic hosts for adult fleas, or exposure to pathogens, some potential *Wolbachia* fitness advantages to females may be expressed.

Interactions with other bacteria could also play a role. In particular, coinfection by *Bartonella* spp., the second most abundant bacteria in wild *S. cleopatrae* (Cohen et al., 2015), which is absent in the laboratory colony of fleas, may lead to *Wolbachia* fitness advantages on their flea hosts, if high *Bartonella* loads damage the fleas. Indeed, Morick et al. (2013) found an indication of fitness reduction in *Xenopsylla ramesi* fleas in the presence of *Bartonella*. Therefore, growing the flea under limited nutritional or high competition conditions or in the presence of other bacteria could potentially reveal “hidden” fitness advantages in this system, as well as in other systems with sex-differences in endosymbiont persistence.

### Hypothesis 3 Is Not Supported: There Is No Indication for Reproductive Manipulation Inducing Female-Biased Sex Ratio

*Wolbachia* presence had no effect on the offspring sex ratio, ruling out the possibility of sex-distorting manipulations, such as MK, IP, and MF, which induce female-biased sex ratio in their host (Werren et al., 2008). The fourth common reproductive manipulation, CI, seems unlikely as well, owing to the absence or near absence of *Wolbachia* in males (Bourtzis et al., 1996; Werren et al., 2008; Richardson et al., 2019). However, since CI is not predicted to cause female-biased sex ratio in arthropods (Werren et al., 2008), we cannot rule out this possibility. Moreover, recent evidence suggests that *Wolbachia* can cause CI or at least partial CI, despite its low densities in males (Noda et al., 2001; Richardson et al., 2019). Interestingly, the evidence for a full CI by male fleas with no detected *Wolbachia* comes from a *Wolbachia*-*Drosophila* model system that exhibited an extreme female-biased *Wolbachia* persistence pattern as in our study system, with 100% prevalence and high *Wolbachia* loads in females (cycle number at detection threshold, Cp 27.4 ± 2.7), and low prevalence (36%) and loads in males (Cp 35.3 ± 2.7). Unfortunately, apart from the two above-mentioned model systems, which show indications for CI (Noda et al., 2001; Richardson et al., 2019), to the best of our knowledge, the possibility of reproductive manipulations has not been explored in the other systems with potential sex-differences in endosymbiont persistence.

### Proposed Underlying Mechanisms for the Persistence of Endosymbionts With Sex-Biased Infection Patterns

Our findings, combined with that of others (Noda et al., 2001; Gorham et al., 2003; Weinert et al., 2007; Richardson et al., 2019), highlight the question: how do endosymbionts persist in systems with extreme sex-biased endosymbiont infection levels? As noted above, CI and fitness advantages for female hosts, under limiting conditions, are two plausible mechanisms that should be further investigated.

An alternative possibility is that past reproductive manipulation or fitness effects allowed the endosymbiont to spread in the host population initially, and the high persistence in females is currently maintained *via* high transmission rates. For example, evidence suggests that in the butterfly *Hypolimnas bolina*, male killing has occurred in the past but no longer operates, while *Wolbachia* still persist at high prevalence in host populations (Charlat et al., 2005). Inspired by the “ghost of competition past” (Connell, 1980), we term this alternative hypothesis as the “ghost of past manipulations or fitness effects.” This hypothesis is supported by theoretical models, showing that a high maternal transmission rate can ensure endosymbiont persistence in the population in the absence of a reproductive manipulation (Prout, 1994; Turelli, 1994; Richardson et al., 2019). Moreover, an evolutionary increase in the transmission rate, at the expense of reproductive manipulation, is consistent with the low endosymbiont prevalence in males. This is because selection to maintain high transmission to males may not be as strong as selection to maintain high transmission to females, considering that males constitute an evolutionary dead-end for the symbiont (Normark, 2004).

In conclusion, we found no evidence for any of the suggested underlying mechanisms for the high endosymbiont persistence in female fleas, calling for additional exploration of hidden fitness effects, the occurrence of CI, or alternative mechanisms, in this particular system, as well as in additional systems with sex-bias differences in endosymbiont persistence. A better understanding of the persistence mechanisms in a variety of systems will shed further light on the evolution and ecology of arthropod-endosymbiont interactions in nature.

## Data Availability Statement

The raw data supporting the conclusions of this article will be made available by the authors, without undue reservation.

## Ethics Statement

The handling protocol was approved by the committee for the ethical care and use of animals in experiments of Ben-Gurion university of the Negev (IL-85-10-2019b).

## Author Contributions

All authors contributed to the concept and design of the study. RF established the cured and infected line and conducted the feeding and reproduction experiment and all the molecular analyses. RF and HH performed the statistical analysis. All authors contributed to the manuscript preparation and read and approved the submitted version.

### Conflict of Interest

The authors declare that the research was conducted in the absence of any commercial or financial relationships that could be construed as a potential conflict of interest.
